# Reference-based checklist of gilled Agaricales (Basidiomycota, Fungi) from Ukraine

**DOI:** 10.3897/BDJ.11.e99101

**Published:** 2023-01-11

**Authors:** Oleh Prylutskyi, Mykola Prydiuk, Vasyl Malanyuk, Valeria Yakunina

**Affiliations:** 1 V.N. Karazin Kharkiv National University, Kharkiv, Ukraine V.N. Karazin Kharkiv National University Kharkiv Ukraine; 2 M.G. Kholodny Institute of Botany NAS of Ukraine, Kyiv, Ukraine M.G. Kholodny Institute of Botany NAS of Ukraine Kyiv Ukraine; 3 Halych National Nature Park, Halych, Ukraine Halych National Nature Park Halych Ukraine

**Keywords:** agarics, checklist, Ukraine

## Abstract

**Background:**

Agaricales is the largest order within the class Agaricomycetes (Basidiomycota, Fungi). Most genera have a gilled (lamellate) basidiomata, though gasteroid, secotioid and cyphelloid fruit bodies also occur in several families and genera. Although gilled Agaricales (usually called "agarics") are the most investigated part of the Fungi, the last summary of their diversity in Ukraine was published back in 1996 and needs to be updated. Only several families have undergone an in-depth review over the last 30 years. Most of the data on species occurrences distributed throughout Ukraine are only partially digitised, under-represented on the Web and published primarily in Ukrainian sources.

**New information:**

Here, we provide the list of the 1201 scientific names of gilled Agaricales (species and infraspecific taxon ranks) ever reported from the territory of Ukraine, based on the more than 300 sources published from 1900 to 2021, as well as digitised collection specimens from three mycological collections. For each taxon mentioned in the checklist, we provide references to either known collection specimens or published sources, where researchers can find more information about the records – 8797 records in total.

## Introduction

Agaricales is the largest order within the class Agaricomycetes (Basidiomycota, Fungi). Most genera have a gilled (lamellate) basidiomata, though gasteroid, secotioid and cyphelloid types of fruit bodies also occur in several families and genera.

Gilled Agaricales have attracted the attention of mycologists studying fungal diversity in the current territory of Ukraine since the 19^th^ century. However, the first attempt to compile the checklist for its representatives known from the territory of Ukraine was published back in 1979 in the "Handbook of Fungi of Ukraine" (Zerova et al. 1979). Unfortunately, this source did not provide references to the specimens or at least exact dates and places of records, but only botanical regions from which species were reported.

In the 1980s, "Flora Fungorum Ucrainicae" was launched, aimed to provide critical revisions for the major groups of Ukrainian fungi, with references to particular records. To date, three volumes covering gilled Agaricales have been published, devoted to Agaricaceae (Wasser 1980), Amanitales (currently included into Agaricales as Amanitaceae and Pluteaceae, Wasser (1992)), Bolbitiaceae and Coprinaceae (currently Psathyrellaceae, Prydiuk (2015)). The last summary of the known diversity of gilled Agaricales of Ukraine, amongst other fungi, was published in 1996 (Minter and Dudka 1996). Only a part of the mentioned publication was digitised and only the last one is available online (Andrianova et al. 2006). Only a tiny fraction of the data on Ukrainian gilled Agaricales is open access, both human- and machine-readable and meets modern biodiversity data standards (Wieczorek et al. 2012), such as a digitised part of the Mycological collection of the Herbarium of V.N. Karazin Kharkiv National University (Savchenko et al. 2020).

Here, we provide the list of the 1201 scientific names of gilled Agaricales reported from the territory of Ukraine (species and infraspecific taxa), based on more than 300 sources published from 1900 to 2021, as well as digitised collection specimens from three mycological collections. For each taxon mentioned in the checklist, we provide references to either known collection specimens or published sources, where researchers can find more information about the records – 8797 records in total. Data were published as a "checklist with occurrences" dataset (Prylutskyi et al. 2022) and accessible via GBIF.org under the Creative Commons Zero v.1.0 Universal licence.

## Project description

### Title

Northern Eurasia 2022

## Sampling methods

### Study extent

Data for this checklist comprise references from 345 published sources issued from 1900 to 2021. We also incorporated information on digitised collection specimens from three mycological collections – the mycological part of the Herbarium of M.G. Kholodny Institute of Botany of National Academy of Sciences of Ukraine, Kyiv (KW-M), the mycological part of the Herbarium of V.N. Karazin Kharkiv National University, Kharkiv (CWU(MYC)) and the mycological collection of Halych National Nature Park, Halych (VM(MYC)) – totalling 5371 specimens. Specimens and references were included based on the following criteria:

1. Taxon belongs to the Order Agaricales (excluding families Clavariaceae, Cyphellaceae, Fistulinaceae, Niaceae, Phelloriniaceae, Pterulaceae, Schizophyllaceae, Stephanosporaceae, Typhulaceae, as well as genera *Lycoperdon*, *Calvatia*, *Disciseda* and other genera comprising taxa with gasteroid, secotioid and cyphelloid fruit bodies) and identified to either species or infraspecific level.
2. Occurrences lie within the official state boundary of Ukraine.
3. Literature source is credible, i.e. authored by recognised mycologists and published in the peer-review scientific literature (journal articles, monographs, conference proceedings).

### Sampling description

The largest source of the data was the specimens and literature references available through the online database "Fungi of Ukraine" (Andrianova et al. 2006). The whole database comprises data on more than 52,400 records of fungi and fungus-like protists, covering the vast majority of specimens from the KW-M collection, as well as the records from literature sources published by the year 2000. Since data are not downloadable and no longer accessible in a raw format, they were mined semi-automatically, using a custom Python parser written by Valeria Yakunina, for occurrences and literature references separately. Five thousand and seventy-eight occurrences of gilled Agaricales were then filtered, based on family and genus names. Literature sources published after 2000, as well as the sources not covered by "Fungi of Ukraine", such as the "Handbook of Fungi of Ukraine" (Zerova et al. 1979), were digitised manually by the authors of the dataset. Newer collection specimens from the CWU(MYC) and VM(MYC) collections were digitised manually. Specimens from CWU(MYC) collections have also been uploaded into the PlutoF biodiversity data management system (Abarenkov et al. 2010), where they are stored alongside extended information. If the specimen had been mentioned in one of the digitised literature sources, we treated such cases as a single record to avoid duplications.

For georeferenced records from the "Fungi of Ukraine" database, we converted geographic coordinates provided by the source into a decimal format using the formula "degree + minutes/60 + seconds/3600". Since we have no information about georeferencing protocol which has been used during the data preparation, we left terms describing georeference and coordinate uncertainty empty. Records derived from the recent sources were georeferenced by the authors of the dataset either manually from maps or obtained from GPS coordinates when available. Coordinate uncertainty values were calculated following DarwinCore recommendations and Georeference best practices (Chapman and Wieczorek 2020). For the records for which the source provides location information at a level coarser than a particular protected area (e.g. administrative or natural region of Ukraine or accompanied with the remark "Throughout the territory of Ukraine"), we left all georeferencing terms empty.

To make a list of taxa, we harmonised scientific names, provided by the authors of corresponding publications and/or specimens. In the first step, we matched our list of names with the GBIF Backbone Taxonomy (GBIF Secretariat 2022) using GBIF species matching tool, then manually checked mismatching records against Index Fungorum nomenclatural database (Kirk and Cooper 2022). For the names treated as accepted by both sources, we used the name provided by GBIF Backbone Taxonomy. For most of the mismatched names, we followed the Index Fungorum's view. We also kept all the names for infraspecific taxa, represented non-type varietas/forma and followed Index Fungorum in nomenclature for such cases. For some taxa, which both GBIF Backbone Taxonomy and Index Fungorum treat as synonyms, we kept names provided by the authors of the records, for example, *Agaricustabularis* Peck. For each name in the resulting list of scientific names (species, varietas and formas), we then assigned a unique identifier (taxonID), through which taxa linked to the records in the occurrence part of the data. Full scientific names as the authors wrote them were kept in the "verbatimIdentification" column of the "occurrence" data sheet.

### Quality control

Since the primary purpose of this checklist is to provide researchers with the most comprehensive list of the scientific names of gilled Agaricales reported from the territory of Ukraine, we did not make a deep taxonomical revision of the data. That is why synonyms or even ambiguous scientific names might be present. For each taxon mentioned in the checklist, we provide references to either known collection specimens or published sources, where researchers can find more information about the records. We used GBIF species matching tool to find possible typos in scientific names. We also used Index Fungorum nomenclatural database (Kirk and Cooper 2022) to check the current status of names. For data cleaning and final adjustment, we used OpenRefine and R (R Core Team 2022).

### Step description

1. Parsing the "Fungi of Ukraine" database, coordinate conversion into decimal degrees when available.
2. Manual digitisation of the sources either published after the year 2000 or not covered by the "Fungi of Ukraine" database.
3. Extraction of available collection data from CWU(MYC), VM(MYC) and KW-M custom collection management systems.
4. Georeferencing of the records accompanied with annotations of the described location at the level of the particular protected areas or finer.
5. Adaptation of the data to the DarwinCore standard.
6. Nomenclatural revision, preparing the list of scientific names.

## Geographic coverage

### Description

Data cover all the territory of Ukraine. It was possible to georeference 5904 records (67 per cent of the records).

### Coordinates

43.835 and 53.015 Latitude; 21.445 and 41.309 Longitude.

## Taxonomic coverage

### Description

**Figure 1.**
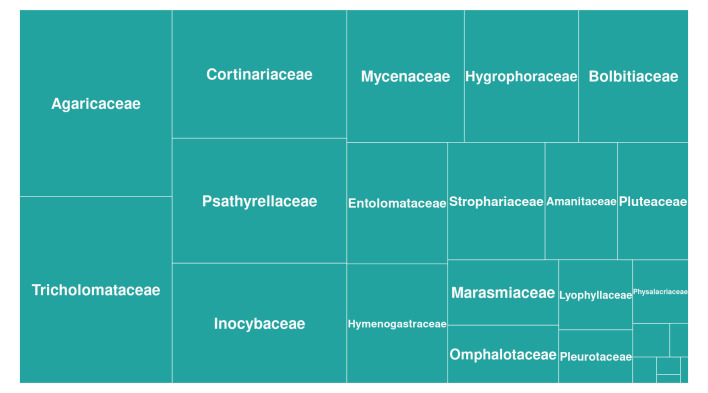
Taxonomic structure of gilled Agaricales reported from Ukraine. The area of rectangles corresponds with the relative number of species and infraspecific taxa within a particular family. The richest families were Tricholomataceae (136 species and infraspecific taxa), Agaricaceae (130 taxa), Cortinariaceae and Psathyrellaceae (each included 104 taxa) and Inocybaceae (96 species and infraspecific names). Families Hydnangiaceae, Tubariaceae, Porotheleaceae and Clavariaceae included fewer than ten species each and were not labelled in the Figure.

Order Agaricales (Basidiomycota, Agaricomycetes, Fungi), excluding families Clavariaceae, Cyphellaceae, Fistulinaceae, Niaceae, Phelloriniaceae, Pterulaceae, Schizophyllaceae, Stephanosporaceae and Typhulaceae, as well as genera *Lycoperdon*, *Calvatia*, *Disciseda* and other genera comprising taxa with gasteroids, secotioids and cyphelloids fruit bodies. In total, there were 1201 species and infraspecific taxa names belonging to the 23 families and 172 genera of Agaricales (Fig. 1).

### Taxa included

**Table:** 

Rank	Scientific Name	
kingdom	Fungi	
phylum	Basidiomycota	
class	Agaricomycetes	
order	Agaricales	

## Temporal coverage

### Notes

1900 –2022

## Collection data

### Collection name

M.G. Kholodny Institute of Botany, National Academy of Sciences of Ukraine Herbarium (KW-M); V. N. Karazin National University Herbarium (CWU(MYC)); Mycological collection of the Halych National Nature Park, Halych (VM(MYC))

### Collection identifier

http://grscicoll.org/institution/national-academy-sciences-ukraine; http://grscicoll.org/institution/v-n-karazin-kharkiv-national-university

### Specimen preservation method

dried

## Usage licence

### Usage licence

Creative Commons Public Domain Waiver (CC-Zero)

### IP rights notes

To the extent possible under law, the publisher has waived all rights to these data and has dedicated them to the Public Domain (CC0 1.0). Users may copy, modify, distribute and use the work, including for commercial purposes, without restriction.

## Data resources

### Data package title

Reference-based checklist of gilled Agaricales (Basidiomycota, Fungi) from Ukraine

### Resource link

https://www.gbif.org/dataset/f994f6e8-4d7d-45bd-ab10-d59ecdfdbe80

### Alternative identifiers

https://doi.org/10.15468/bgv8hy

### Number of data sets

1

### Data set 1.

#### Data set name

Reference-based checklist of gilled Agaricales (Basidiomycota, Fungi) from Ukraine

#### Data format

Darwin Core

#### Character set

UTF-8

#### Download URL

https://www.gbif.org/dataset/f994f6e8-4d7d-45bd-ab10-d59ecdfdbe80

#### Description

The data part of the Darwin Core Archive includes two tabulation-delimited tables, linked by the values in "taxonID" field:

- taxon.txt, with 13 fields in Darwin Core terms and 1,202 records, provides information on scientific names, rank and higher classification for each species or infraspecific taxa.
- occurrence.txt, with 28 fields in Darwin Core terms and 8,797 records, provides information for occurrences, preserved specimens and references for each taxon listed in the taxon.txt file.

**Data set 1.** 

Column label	Column description
taxonID (Darwin Core Taxon, Darwin Core Occurrence Extension)	Unique identifier of each taxon (species, forma or varietas) listed in the Taxon Core. Type form/varietas treated as species.
scientificName (Darwin Core Taxon)	Binary name of the species (genus + specific epithet), forma or varietas (genus + specific epithet + infraspecific epithet).
higherClassification (Darwin Core Taxon)	Concatenated list of hig.her taxa, from kingdom to genus.
kingdom (Darwin Core Taxon)	The full scientific name of the kingdom in which the taxon is classified.
phylum (Darwin Core Taxon)	The full scientific name of the phylum in which the taxon is classified.
class (Darwin Core Taxon)	The full scientific name of the class in which the taxon is classified.
order (Darwin Core Taxon)	The full scientific name of the order in which the taxon is classified.
family (Darwin Core Taxon)	The full scientific name of the family in which the taxon is classified.
genus (Darwin Core Taxon)	The full scientific name of the genus in which the taxon is classified.
specificEpithet (Darwin Core Taxon)	The name of the species epithet of the scientificName.
infraspecificEpithet (Darwin Core Taxon)	The name of the infraspecific epithet of the scientificName for either formas or varietas.
taxonRank (Darwin Core Taxon)	The taxonomic rank of the most specific name in the scientificName.
scientificNameAuthorship (Darwin Core Taxon)	Full nomenclatural citation for scientific name.
occurrenceID (Darwin Core Occurrence Extension)	An identifier of a particular occurrence, unique within Occurrence extension data table.
verbatimIdentification (Darwin Core Occurrence Extension)	Original scientific names as provided in either publication or specimen label to which an Occurrence referenced.
basisOfRecord (Darwin Core Occurrence Extension)	The method in which data were acquired. Three levels: “PreservedSpecimen” for Occurrences derived from collection specimens, “MaterialCitation” for Occurrences derived from scholarly publications and “HumanObservation” for the Occurrences obtained from field diaries and stored in “Fungi of Ukraine” database.
institutionCode (Darwin Core Occurrence Extension)	Character code of the institution keeping mycological collections.
collectionCode (Darwin Core Occurrence Extension)	Character code (acronym) of the collection.
catalogNumber (Darwin Core Occurrence Extension)	Unique identifies of specimen within a collection.
verbatimEventDate (Darwin Core Occurrence Extension)	The time of the Occurrence was made as it might be extracted from the publication. In some cases – as date ranges.
eventDate (Darwin Core Occurrence Extension)	The full date of the Occurrence according to the Darwin Core date format recommendations. Many sources we used did not contain precise information about the date of each Occurrence, only the overall research time-window. Since intervals cannot be reduced to any particular date, GBIF.org automatically downscales such intervals to the 1st Jan of the first Year of the time-window, which may be misleading for the people referenced directly to the GBIF.org portal. Please download our data as DarwinCore archive, which contains dates as we put them. Any user’s download of those data, including search query results, will return true dates. Moreover, GBIF.org displays full information from all the fields for each record by clicking on it (example occurrence: https://www.gbif.org/occurrence/3986574311).
eventRemarks (Darwin Core Occurrence Extension)	Information on how the verbatimEventDate was obtained: “date from annotation” for cases when it was possible to derive a date or date range for each Occurrence or “date from a year of publication” for cases when it was not possible. Left blank for occurrences derived from the sources that only mentioned the presence of some taxa and did not provide any information about when they were recorded.
recordedBy (Darwin Core Occurrence Extension)	A person or group of people who were explicitly stated as the authors of the Occurrence at the data source.
identifiedBy (Darwin Core Occurrence Extension)	A person or group of people who were explicitly stated as the identifiers of the Occurrence at the data source.
locality (Darwin Core Occurrence Extension)	Verbal description of the locality as it was provided in source.
country (Darwin Core Occurrence Extension)	Country within which the Occurrence was made. One value – Ukraine.
countryCode (Darwin Core Occurrence Extension)	International code of the country within which the Occurrence was made. One value – UA.
stateProvince (Darwin Core Occurrence Extension)	The name of the administrative region of Ukraine in which the Location occurs (name of the administrative region (Oblast) or Autonomous Republic of Crimea or Kyiv City).
verbatimLatitude (Darwin Core Occurrence Extension)	The geographic latitude as it was mentioned in the source.
verbatimLongitude (Darwin Core Occurrence Extension)	The geographic longitude as it was mentioned in the source.
verbatimCoordinateSystem (Darwin Core Occurrence Extension)	Coordinate reference system used for verbatim coordinates.
decimalLatitude (Darwin Core Occurrence Extension)	The geographic latitude in decimal degrees.
decimalLongitude (Darwin Core Occurrence Extension)	The geographic longitude in decimal degrees.
coordinateUncertaintyInMeters (Darwin Core Occurrence Extension)	The distance (in metres) from the given decimalLatitude and decimalLongitude describing the smallest circle containing the whole of the Location. Set as 100 m for GPS coordinates obtained before 05-05-2020, 30-50 m for GPS coordinates obtained since 05-05-2020 and from 200 to 6000 m for the coordinates georeferenced based on the description.
geodeticDatum (Darwin Core Occurrence Extension)	The geodetic datum upon which the geographic coordinates were given.
georeferencedBy (Darwin Core Occurrence Extension)	A person who georeferenced an Occurrence.
georeferenceProtocol (Darwin Core Occurrence Extension)	Description of the method used to determine coordinates.
associatedReferences (Darwin Core Occurrence Extension)	Bibliographic references associated with the Occurrence.
language (Darwin Core Occurrence Extension)	A language of the resource. en for English, uk for Ukrainian.
references (Darwin Core Occurrence Extension)	References to online resources related to the Occurrences.
